# Editorial: Recent advances in minimally invasive thoracic surgery

**DOI:** 10.3389/fsurg.2023.1182768

**Published:** 2023-03-24

**Authors:** Yojiro Yutaka, Calvin Sze Hang Ng

**Affiliations:** ^1^ Department of Thoracic Surgery, Kyoto University Hospital, Kyoto, Japan; ^2^ Division of Cardiothoracic Surgery, The Chinese University of Hong Kong, Hong Kong SAR, China

**Keywords:** minimally invasive thoracic surgery, video assisted thoracic surgery (VATS), robot - assisted thoracic surgery, uniportal VATS (U-VATS), radiofrequency identification (RFID)

**Editorial on the Research Topic** Recent advances in minimally invasive thoracic surgery

## Introduction

Minimally invasive thoracic surgery (MITS) has drastically improved over the past three decades since the first report of video-assisted thoracoscopic surgery (VATS) by Lewis et al. in 1992 (1). Compared with traditional thoracotomy, VATS offers considerable benefits to patients by being less invasive, and it is globally used as a diagnostic and therapeutic tool for a variety of conditions within the chest cavity. The concept of less invasive surgery to greatly reduce the trauma of chest surgery and preserve lung volume led to the development of robotic-assisted thoracic surgery (RATS) or uniportal VATS, and evidence supporting the utility of sublobar resection for early-stage lung cancer has been established (2, 3). Further technological advances have made it possible to perform VATS or RATS *via* a single port. Compared with traditional thoracic procedures through thoracotomy, these MITS techniques are technically feasible, and they will undoubtedly offer considerable benefits to patients in the future. However, the surgeon must control these various techniques or instruments, and caution should be employed when introducing these novel techniques in appropriately selected patients with lung cancer to ensure long-term survival is not compromised. Therefore, further investigations are needed to understand the recent advances in MITS (4). In this section, we shared new insights into the latest techniques currently available in minimally invasive surgery, and the key limitations or aspects requiring improvement for the future were discussed.

Thanks to coworkers who contributed research topics regarding recent advances in MITS, 12 articles were collected in this edition: seven original studies, two review articles, and three case reports.

Ling Wang et al. reported postoperative diaphragmatic hernia following thoracoscopic sympathectomy for primary palmar hyperhidrosis. RATS has allowed surgeons to perform precise procedures with more flexibility during the operation with an enhanced 3D visualization system through smaller incisions compared with conventional open surgery and VATS. However, the visual field is rather limited by the enhanced magnification, and careful attention should be paid to the possibility of injury to other organs outside the field of vision (5).

Two reviews compared RATS for esophagectomy and thymoma resection with conventional open approaches using meta-analysis, and RATS might be accepted when it is oncologically feasible. There is no established evidence from high-quality randomized controlled trials concerning the clinical difference between conventional approaches and RATS. We should obtain essential information regarding MITS beyond the feasibility of surgical techniques.

In this research topic, more advanced techniques using uniportal RATS were reported. Edoardo Mercadante et al. reported uniportal RATS lobectomy using three robotic arms of the da Vinci Xi system. The key to the successful introduction of this approach was to avoid potential fighting between the robotic arms. Bo Yang et al. reported an initial successful experience of single-port RATS for mediastinal tumors using the da Vinci SP system with a flexible double-jointed instrument that ensures that the lens does not conflict with the two Endowrists.

Both authors demonstrated the successful introduction of uniportal RATS through a smaller incision, although careful case selection and preoperative planning should be performed prior to these surgeries. In the near future, these approaches will become advantageous for surgeries requiring wide operative fields including esophagectomy and lung segmentectomy. However, for the standardization and global introduction of uniportal RATS, further technological advancements including the development of new staplers or suturing devices will be required, and additional clinical evidence should be established regarding long-term oncological outcomes from well-designed randomized trials of this technique.

Overall, these valuable contributions provided important information that could be helpful for the introduction of new technology in MITS. Because the methods reported in this topic, including our wireless localization techniques without lung palpation for small faint pulmonary lesions, are relatively new, additional studies in the future will likely improve the efficacy and safety of these techniques (6, 7). Although we should have an open mind regarding these innovative approaches, appropriate evaluation will be required for the introduction of new techniques or technologies, and treatment should be tailored to each patient to optimize outcomes. The environments surrounding MITS will undergo further development by experienced surgeons. We hope that this supplemental issue of “Recent Advances in Minimally Invasive Thoracic Surgery” will broaden perspectives for thoracic surgeons aiming to improve patient outcomes.
